# CRISPR-Cas12a nucleases function with structurally engineered crRNAs: SynThetic trAcrRNA

**DOI:** 10.1038/s41598-022-15388-z

**Published:** 2022-07-16

**Authors:** D. J. Jedrzejczyk, L. D. Poulsen, M. Mohr, N. D. Damas, S. Schoffelen, A. Barghetti, R. Baumgartner, B. T. Weinert, T. Warnecke, R. T. Gill

**Affiliations:** 1 Novo Nordisk Foundation Center for Biosustainability, Technical University of Denmark, Kemitorvet 220, 2800 Kongens Lyngby, Denmark; 2 Artisan Bio, 363 Centennial Parkway, Suite 310, Louisville, CO 80027 USA

**Keywords:** Genetic engineering, Gene targeting, Biotechnology, Immunology

## Abstract

CRISPR-Cas12a systems are becoming an attractive genome editing tool for cell engineering due to their broader editing capabilities compared to CRISPR-Cas9 counterparts. As opposed to Cas9, the Cas12a endonucleases are characterized by a lack of trans-activating crRNA (tracrRNA), which reduces the complexity of the editing system and simultaneously makes CRISPR RNA (crRNA) engineering a promising approach toward further improving and modulating editing activity of the CRISPR-Cas12a systems. Here, we design and validate sixteen types of structurally engineered Cas12a crRNAs targeting various immunologically relevant loci *in-vitro* and *in-cellulo*. We show that all our structural modifications in the loop region, ranging from engineered breaks (STAR-crRNAs) to large gaps (Gap-crRNAs), as well as nucleotide substitutions, enable gene-cutting in the presence of various Cas12a nucleases. Moreover, we observe similar insertion rates of short HDR templates using the engineered crRNAs compared to the wild-type crRNAs, further demonstrating that the introduced modifications in the loop region led to comparable genome editing efficiencies. In conclusion, we show that Cas12a nucleases can broadly utilize structurally engineered crRNAs with breaks or gaps in the otherwise highly-conserved loop region, which could further facilitate a wide range of genome editing applications.

## Introduction

CRISPR-Cas endonuclease editing systems provide a robust platform for genetic manipulation, which holds great potential for various applications in biotechnology and medicine, most notably cancer immunotherapy for the treatment of various malignancies^1–7^. The type V-A nuclease Cas12a, formerly known as Cpf1, which targets sequences with T-rich protospacer adjacent motifs (PAMs), is guided by a single CRISPR RNA (crRNA) complementary to the targeted DNA sequence, and lacks a separate trans-activating crRNA (tracrRNA)^8–11^. Cas12a crRNAs are typically 42 nucleotides long, of which 21–25 nucleotides in the crRNA 3’-end are variable and guide the nuclease by sequence-specific hybridization to targeted duplex DNA. A highly-conserved stem-loop structure at the 5’-end of the crRNA is essential for nuclease recognition and enzymatic function^11^.

Much effort has been applied to improve the editing capabilities of CRISPR-Cas systems^9,12,13^ as well as the discovery of novel Cas12a-family nucleases^11,14,15^. Engineering of crRNAs is indubitably an attractive approach to design novel functions due to the low cost of polynucleotide synthesis and advances in the understanding of polynucleotide function. crRNAs have been modified to improve editing in a number of ways, namely, by adjusting the length of the 3’ or 5’ regions^16,17^, removing majority of the hairpin scaffold^18^, introducing chemical or structural modifications^19–22^, or inserting tRNA-like structures or small hairpins in the 3’-end to increase stability^23^. Li et al*.* (2017) tested splitting the highly-conserved stem-loop region at a single site and observed a complete loss of gene-cutting activity in the presence of Cas12a nuclease^19^. Lastly, shortening the DNA complementary fragment (spacer) of crRNAs has been shown to reduce off-target editing without compromising the editing efficiency^24^.

The classification of CRISPR-Cas systems remains challenging because they show remarkable diversity in terms of gene composition, genomic locus architecture, and low sequence similarity, even in the core genes shared by many CRISPR-Cas variants^25^. Due to the constant evolution of CRISPR-Cas systems and frequent shuffling of the adaptation and effector modules, current classification is based on combined, semi-formal criteria, such as the presence and amino acid sequence of signature *cas* genes, the presence, phylogeny and organization of the genes in the CRISPR-cas loci^25,26^, and the configuration of the guide RNAs. In addition, the subtype classification of CRISPR-Cas systems further contributes to the overall classification complexity, as it requires characterization of the signature genes or, in their absence, more detailed analysis of the sequence profiles^26^.

Therefore, the diversity of configurations observed in such CRISPR-Cas systems that can be exploited to engineer novel synthetic systems, exhibiting improved or altered performance, may be numerous. Here, we hypothesized that the wild-type Cas12a (type V-A) single-stranded crRNAs could be split into fully functioning tracrRNAs-like and crRNAs-like molecules by introducing engineered breaks or large gaps in the highly-conserved crRNA loop region while retaining the gene editing efficiency in the presence of Cas12a nucleases. Further, we sought to test the extent to which any observed Cas12a tolerance to SynThetic trAcrRNAs (STARs) could be employed as the basis for a facile strategy to alter, improve, and/or control the function of the editing systems.

## Results

**Figure 1:**
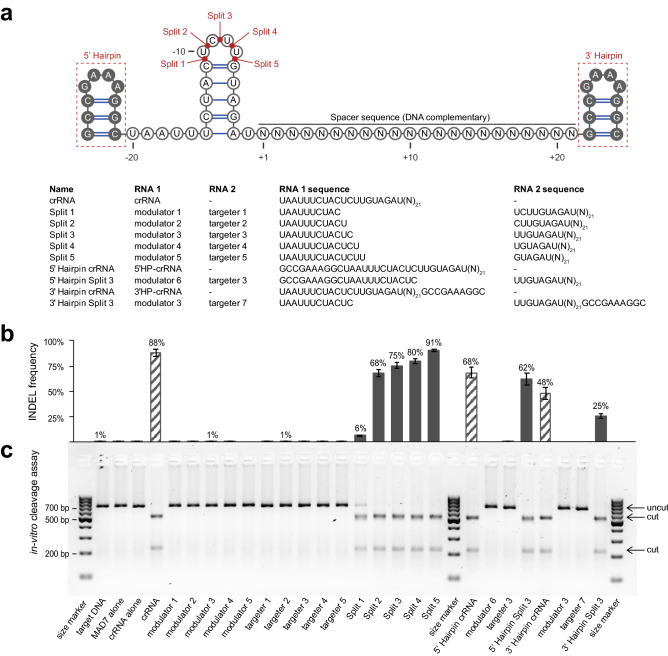
SynThetic trAcer RNAs (STARs) modulate gene editing in mammalian cells. (**a**) Structure and sequence of SynThetic trAcer RNAs (STARs). (**b**) INDEL frequency (%) of MAD7 with wild-type crRNA, individual STAR components, and Split STAR-crRNAs targeting the DNMT1 locus, measured by amplicon sequencing (error bars: mean ± SEM for n ≥ 3). (**c**) *In-vitro* cleavage assay on the amplicon containing the DNMT1 locus target sequence, size marker: GeneRuler 100 bp DNA Ladder (ThermoFisher Scientific), cropped image. Original gel image is presented in Extended Data (**c**). The sequences of all crRNAs used are listed in Supplementary Table 1.

To test the gene-cutting ability of Cas12a nucleases with split crRNAs, we systematically designed and synthesized an array of STARs where the existing synthetic crRNA is split within the loop region into two separately synthesized RNAs (Split 1–5) (Fig. 1a). The 5’ short (~ 12nt) fragment mimics the 5’-handle of the wild-type crRNA. Changes in this 5’ fragment could be used to modulate activity of the overall editing system, and thus referred to as the “modulator” RNA. The 3’ fragment comprises the nucleotides responsible for hybridization to the targeted DNA-duplex and is referred to as the “targeter” RNA (Fig. 1a). In addition, we designed STARs and crRNAs that were modified with an additional hairpin secondary structure on the 5’- or 3’-end (Fig. 1a).

To test our STAR-crRNAs, we used the recently described Cas12a nuclease, ErCas12a, also known as MAD7^15,27^, and the ribonucleoprotein (RNP) based editing strategy for *in-cellulo* studies^28^. Using both *in-vitro* cleavage reactions and *in-cellulo* insertion and deletion (INDEL) assays in human leukemic T-cells (Jurkat), we found that crRNAs that are split at various locations within the loop retained the ability to guide MAD7 nuclease activity to the DNMT1 gene, while neither the modulator nor the targeter RNAs were sufficient alone (Fig. 1b-c). Split STAR-crRNAs showed comparable DNA cleavage activity to unmodified crRNAs, except for Split 1 and 3’ Hairpin Split 3 crRNAs. It is important to note that the observed DNA cleavage was not due to the excess of MAD7-RNP amounts, which was confirmed by the serial dilution experiments *in-vitro* and *in-cellulo* (Supplementary Figs. 1a-b). We used Sanger sequencing to determine the position of DNA cleavage by MAD7 and to determine whether it was altered by use of Split STAR-crRNAs (Supplementary Fig. 1c). Similar to the DNA strand cleavage position previously reported for FnCas12a, AsCas12a, and LbCas12a^11^, MAD7 cleaved after the 18^th^ nucleotide on the non-targeted strand and the 23^rd^ nucleotide on the targeted strand. Notably, we found no substantial difference in the position of DNA cleavage for unmodified crRNAs and engineered STAR-crRNAs.

**Figure 2:**
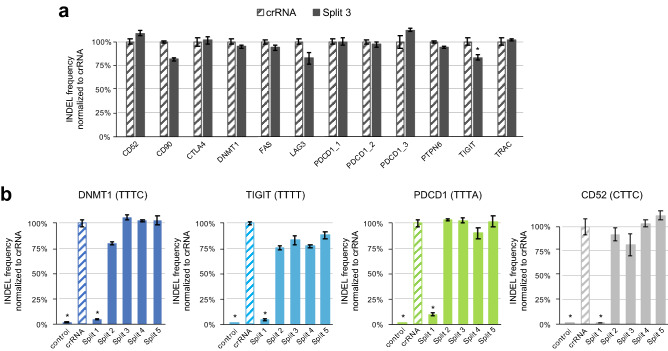
SynThetic trAcer RNAs (STARs) editing efficiency at immunologically important target sites. (**a**) INDEL frequency (%) of MAD7 with wild-type crRNA and Split 3 STAR-crRNA targeting twelve selected loci, measured by amplicon sequencing (error bars: mean ± SEM for n = 6). Unnormalized INDEL frequency (%) data are presented in Supplementary Fig. 2a. (**b**) INDEL frequency (%) of MAD7 with wild-type crRNA and Split STAR-crRNAs targeting the DNMT1, TIGIT, PDCD1, and CD52 loci with different PAM sequences (TTTC, TTTT, TTTA, and CTTC respectively), measured by amplicon sequencing (error bars: mean ± SEM for n ≥ 3). Unnormalized INDEL frequency (%) data are presented in Supplementary Fig. 2b. All samples showing significant changes in INDEL frequencies compared to wild-type crRNAs, $P$ ≤ 0.05 using two-sided *t*-test after a global one-way analysis of variance (ANOVA), are indicated with asterisk. The sequences of all crRNAs used are listed in Supplementary Table 1.

To validate the robustness and genericity of structurally engineered STAR-crRNA molecules, we chose Split 3 STAR-crRNA to ensure the loop disruption. We tested three additional Split 3 constructs with different PAM sequences *in-vitro* (Supplementary Fig. 1d) and twelve Split 3 crRNA targeting immunologically relevant loci *in-cellulo* (Fig. 2a). These results showed that *in-vitro* cleavage required both the modulator and the targeter RNAs, and at the same time verified the robust *in-cellulo* editing of Splits 3 at immune-oncology relevant loci, specifically CD90, CTLA4, FAS, LAG3, PTPN6, and TRAC. Split 3 crRNAs exhibited comparable target DNA cleavage efficiencies to those of their wild-type crRNA counterparts. We further tested all five Split STAR-crRNAs and verified the robust *in-cellulo* editing of Splits 2–5. Similar to editing at the DNMT1 in our preliminary experiments, Split 1 STAR-crRNA showed drastically reduced cleavage activity at all target sites (Fig. 2b).

**Figure 3:**
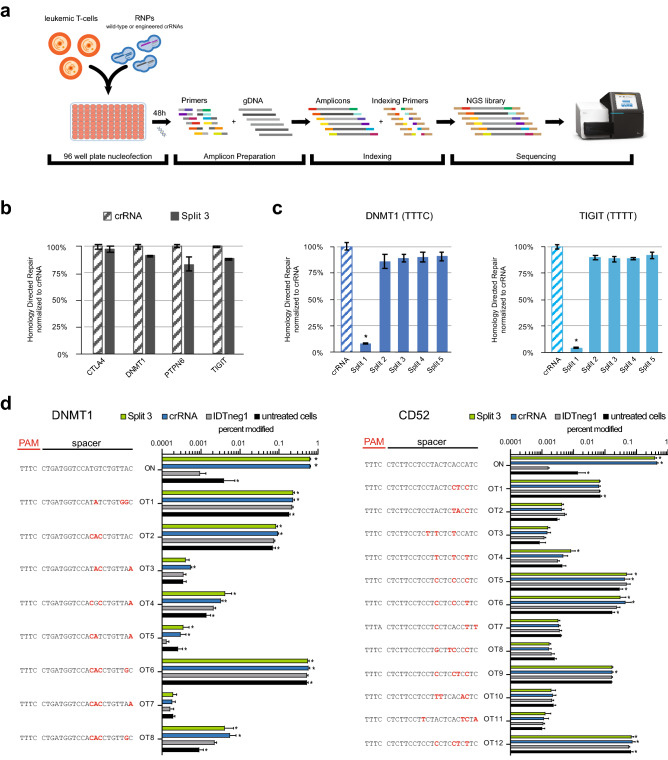
SynThetic trAcer RNAs (STARs) supporting human-cell engineering. (**a**) Schematic representation of the genome editing workflow. (**b**) Successful Homology Directed Repair (HDR, %) of MAD7 with wild-type crRNA and Split 3 STAR-crRNA at the CTLA4, DNMT1, PTPN6, and TIGIT loci, measured by amplicon sequencing (error bars: mean ± SEM for n = 6). Unnormalized HDR (%) data are presented in Supplementary Fig. 3a. (**c**) Successful Homology Directed Repair (HDR, %) of MAD7 with wild-type crRNA and various Split STAR-crRNA targeting the DNMT1 and TIGIT loci, measured by amplicon sequencing (error bars: mean ± SEM for n = 6). Unnormalized HDR (%) data are presented in Supplementary Fig. 3b. (**d**) Off-target INDEL frequency analysis (%) of MAD7 with wild-type crRNA and Split 3 STAR-crRNA targeting the DNMT1 and CD52 loci, measured by amplicon sequencing (error bars: mean ± SEM for n ≥ 3). Wild-type crRNA and Split 3 STAR-crRNA generated significant INDEL modifications at twelve off-target sites ($P$ ≤ 0.05) relative to IDTneg1 crRNA. Hypothesis was tested using a two-sided Fisher exact test with pooled read counts. Panels **b** and **c**: Samples showing significant changes in INDEL frequencies compared to wild-type crRNA, $P$ ≤ 0.05 using two-sided *t*-test after a global one-way analysis of variance (ANOVA), are indicated with asterisk. The sequences of all crRNAs used are listed in Supplementary Table 1.

One of the major goals in cell engineering is to site-specifically insert DNA payloads via Homology-Directed Repair (HDR). To test the effect of Split STAR-crRNAs on HDR, we assayed insertion of short DNA sequences using single-stranded oligonucleotide (ODN) HDR templates (HDRTs). As a means to avoid nuclease re-targeting after HDR, a BamHI restriction enzyme recognition site was introduced into the HDR templates to eliminate both the PAM sequence and the majority of the targeted sequence (Supplementary Table 2). Genome editing was assayed by amplicon sequencing to measure both the frequency of INDEL formation and the efficiency of HDR insertion (Fig. 3a). Our results showed that the efficiency of Split 3 STAR-crRNA was comparable to the wild-type crRNA in driving HDR at four different loci (Fig. 3b). We further tested the efficiency of all five Split STAR-crRNAs at two loci, and observed that, similar to the DNA cleavage and INDEL formation, Splits 2–5 supported HDR insertions at levels comparable to the wild-type crRNAs, while Split 1 exhibited drastically reduced efficiency of HDR insertion (Fig. 3c). We used Split 3 STAR-crRNA to additionally assess whether the engineered break in the crRNA resulted in increased frequency of off-target editing activity. Primer sets were designed to analyze the top twenty off-target sites (≤ 4 mismatches within the crRNA spacer) predicted by CasOFFinder^29^, comparing Split 3 and the corresponding crRNA. Most predicted off-target sites did not exhibit substantially increased INDEL formation, suggesting that these sites are not edited, or are edited at levels that are below the detection limit of our assay (Fig. 3d). However, twelve off-target sites showed statistically significant INDEL formation compared to the samples treated with non-targeting crRNA (IDTneg1, IDT). We observed small but significant off-target activity between the non-targeting crRNA and either wild-type crRNA or Split 3 STAR-crRNA at three off-target sites, OT3 for DNMT1, and OT4 and OT9 for CD52 targeting spacer (Fig. 3d). It is important to note that the observed background editing in both IDTneg1 treated and untreated cells at the OT1 and OT6 sites likely occurred due to the errors in NGS base calling or substitutions arising during DNA amplification.

**Figure 4:**
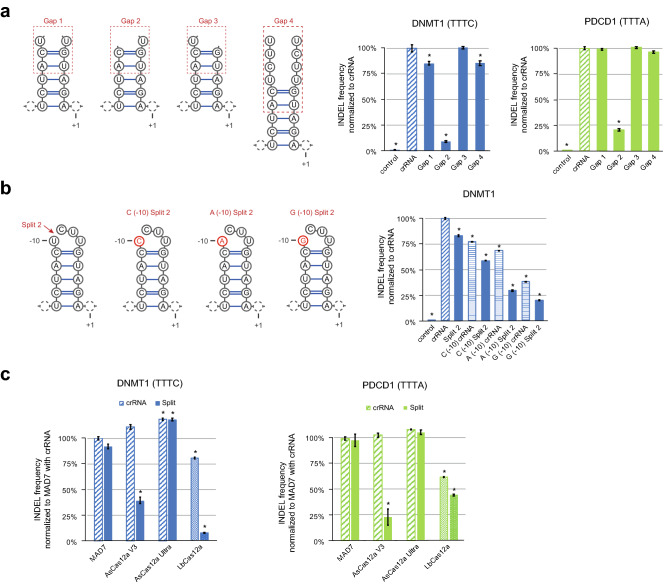
Gap and substituted STARs supporting human-cell engineering. (**a**) INDEL frequency (%) of MAD7 with wild-type crRNA and Gap STAR-crRNAs targeting the DNMT1 and PDCD1 loci, measured by amplicon sequencing (error bars: mean ± SEM for n ≥ 3). (**b**) INDEL frequency (%) of MAD7 with wild-type crRNA and Split 2 STAR-crRNAs substituted at position (− 10) in the loop, targeting the DNMT1 locus, measured by amplicon sequencing (error bars: mean ± SEM for n = 3). (**c**) INDEL frequency (%) of MAD7 and AsCas12a (Cas12a V3 and Ultra, IDT) with wild-type crRNA and Split 3 STAR-crRNAs, and and LbCas12a (EnGen LbaCas12a, NEB) with wild-type Lb-crRNA and Lb-Split 4, targeting the DNMT1 and PDCD1 loci, measured by amplicon sequencing (error bars: mean ± SEM for n ≥ 3). Panels **a**, **b**, and **c**: Samples showing significant changes in INDEL frequencies compared to wild-type crRNAs, $P$ ≤ 0.05 using two-sided *t*-test after a global one-way analysis of variance (ANOVA), are indicated with asterisk. The sequences of all crRNAs used are listed in Supplementary Table 1.

Overall, we observed high tolerance of MAD7 to various Split STAR-crRNAs tested in this study. To further assess the tolerance of MAD7 to altered crRNAs, we next synthesized the modulator and the targeter RNAs for STAR-crRNAs with large gaps followed by supplemental nucleotides (Fig. 4a). These constructs were tested by *in-cellulo* INDEL formation in Jurkat cells using the amplicon sequencing assay. Notably, editing was unaffected by the removal of all nucleotides in the loop region except for the U (-10), the same position required for activity of Split 1 STAR-crRNA (Fig. 4a). In addition, insertion of four additional nucleotides by duplicating the loop sequence (Gap 4) had no discernable impact on INDEL formation, indicating that MAD7 can tolerate insertion of nucleotides in the loop region. Finally, we determined that substitutions of the U (-10) position alone or in combination with Split 2 could be used to modulate editing at the same DNMT1 site in Jurkat cells (Fig. 4b).

These results provide novel insights into the development of simple design rules for modulating MAD7 editing activity. Next, we examined whether the observed tolerance to the STAR-crRNA designs would extend to other Cas12a family nucleases. We assayed both *in-vitro* DNA cleavage and *in-cellulo* INDEL formation using two commercially available variants of AsCas12a (Cas12a-V3 and Cas12a-Ultra, IDT) and commercially available LbCas12a (EnGen LbaCas12a, NEB). We used MAD7 crRNAs to guide both AsCas12a effectors, while for LbCas12a we designed a separate set of its respective crRNAs and STAR-crRNAs. Analysis of *in-vitro* DNA cleavage showed that MAD7, AsCas12a-V3, and AsCas12a-Ultra had comparable cleavage activity using either wild-type crRNAs or engineered STAR-crRNAs (Split 3), while LbCas12a showed reduced activity compared to MAD7 and both AsCas12a (Supplementary Fig. 4a). Interestingly, when assaying the DNMT1 locus in Jurkat cells, both AsCas12a-V3 and LbCas12a showed significantly reduced average INDEL formation when using Split 3 STAR-crRNA relative to their wild-type crRNAs; at the PDCD1 locus, however, this reduction in INDEL formation was greater for AsCas12a-V3 than for LbCas12a. In contrast, MAD7 and AsCas12a-Ultra showed high editing efficiency with both Split 3 STAR and wild-type crRNA (Fig. 4c). This result indicates that not all Cas12a nucleases tolerate loop modifications, and that the two commercially available AsCas12a differ in this respect. Moreover, we observed reduced activity of LbCas12a compared to MAD7 when using the wild-type crRNAs (Fig. 4c). It is important to note that the native LbCas12a crRNAs differ from the AsCas12a and MAD7 crRNAs in both the sequence and length of the crRNA loop (Supplementary Fig. 4b). Use of native LbCas12a crRNAs resulted in INDEL frequencies of 81% and 62% at the DNMT1 and PDCD1 loci, respectively (Fig. 4c), while LbCas12a with MAD7 wild-type crRNAs showed minor activity (Supplementary Fig. 4c). On the other hand, Lb-Split 4 STAR-crRNA designed for LbCas12a led to the marginal INDEL formation efficiency of 8% at the DNMT1 locus but resulted in adequate editing of 44% at the PDCD1 locus compared to MAD7 with crRNA (Fig. 4c and Supplementary Fig. 4c). Next, we tested two other STAR-crRNAs designed for LbCas12a, Lb-Split 1 and Lb-Split 6, observing editing efficiencies < 10% at both target sites (Supplementary Fig. 4c). This indicates that LbCas12a does not tolerate shorter loops and alternate sequences of MAD7 crRNAs, but it may utilize some of the Split crRNAs in a target- or PAM-dependent manner. These observations are in contrast with the previous study^20^, which showed that the LbCas12a activity was eliminated altogether when guided by split crRNA. However, our data suggest that LbCas12a is more conservative than AsCas12a in its interaction with crRNA and less tolerant of crRNA modifications. Notably, MAD7 was able to utilize native LbCas12a crRNAs without affecting INDEL formation (Supplementary Fig. 4d). This is consistent with the observed tolerance to Gap 4 STAR-crRNA (Fig. 4a), highlighting the greater tolerance of MAD7 to altered crRNAs compared to LbCas12a.

**Figure 5:**
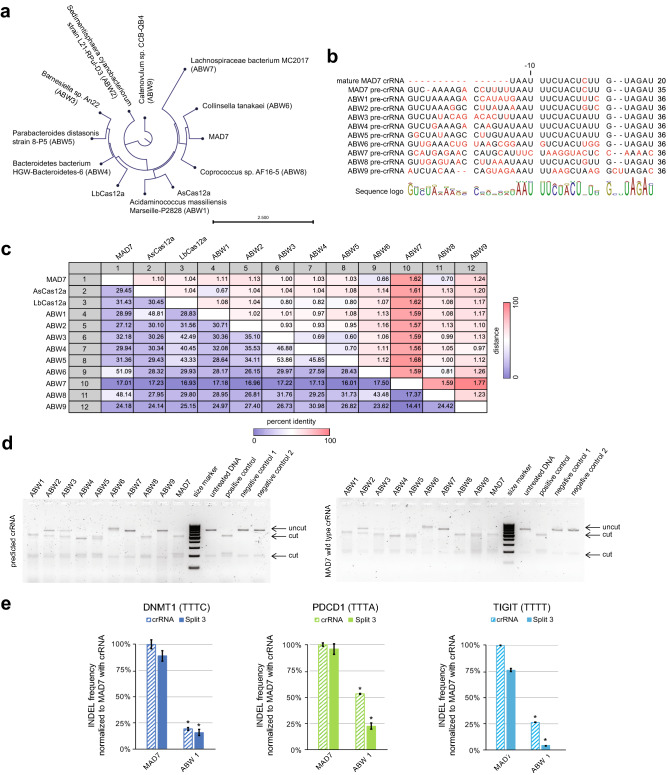
Extension of the STAR system to other class II type-V-A-like nucleases. (**a**) Circular phylogram reflecting evolutionary relationship of MAD7, AsCas12, LbCas12a, and ABW1-9. (**b**) Alignment of the MAD7 and ABW1-9 crRNAs with the mismatches indicated in red. (**c**) Similarity matrix (%) showing the percent identity of MAD7, AsCas12a, LbCas12a, and ABW1-9 amino acid sequences (lower left) and the evolutionary distance (upper right). (**d**) *In-vitro* cleavage assay on the amplicon containing the DNMT1 locus target sequence: nucleases with predicted ABW crRNAs (left) and with MAD7 wild-type crRNA (right), size marker: GeneRuler 100 bp DNA Ladder (ThermoFisher Scientific), cropped image. Original gel image is presented in Extended Data (**d**). (**e**) INDEL frequency (%) of ABW1 and MAD7 with MAD7 wild-type crRNA and Split 3 STAR-crRNA targeting the DNMT1, PDCD1, and TIGIT loci, measured by amplicon sequencing (error bars: mean ± SEM for n ≥ 3). ABW1 samples indicated with asterisk show significant decrease in INDEL frequencies compared to MAD7 with wild-type crRNAs, $P$ ≤ 0.05 using two-sided *t*-test after a global one-way analysis of variance (ANOVA). The sequences of all crRNAs used are listed in Supplementary Table 1.

Given the observed differences in Cas12a tolerance to STAR-crRNAs, we next tested the extent to which our STAR system could be used with novel, divergent Cas12a nucleases. To identify more Cas12a family members, we mined public databases following methodology previously described in Zetsche et al., 2015. We based the search on AsCas12a and MAD7 amino acid sequences and selected nine uncharacterized proteins that met our technical criteria: the presence of CRISPR array in the genome of the organism of origin, the predictable crRNA sequence, and the over 40% GC content in the coding sequence. We further examined the evolutionary relationship of the nine putative Cas12a—from here onwards ABW1-9^30^—and known Cas nucleases used in this study (Fig. 5a) and aligned amino acid sequences (Fig. 5c). Both dendrogram and sequence similarity matrix suggest that the selected proteins come from diverse bacterial strains and share as little as 15% sequence identity. Alignment of predicted direct-repeat sequences, containing pre- and crRNAs, revealed remarkably conservative sequence of the stem and loop structure directly preceding spacer (Fig. 5b). We ran small-scale synthesis of the nine ABW nucleases, which we tested in the *in-vitro* cleavage assay with the predicted, native pre-crRNAs, and MAD7-optimized crRNA (Fig. 5d). Six ABWs showed cleaving activity with their predicted crRNAs, while seven nucleases cleaved oligonucleotides amplified from the DNMT1 target site when guided by MAD7 crRNA. Finally, using the *in-cellulo* INDEL assay in Jurkat cells, we tested genome editing capacity of ABW1 at the DNMT1, PDCD1, and TIGIT loci with both MAD7 wild-type crRNA and Split 3 STAR-crRNA (Fig. 5e). While ABW1 tolerated the split within the loop, its activity varied in a target- or PAM-dependent manner. The assayed nuclease was both less active and led to lower INDEL formation frequency than MAD7 with both crRNAs (Fig. 5e).

## Discussion

In this study, we explored and tested CRISPR-Cas12a-based editing systems. We hypothesized that split constructs, i.e. STAR-crRNA (SynThetic trAcrRNA), may affect editing by altering affinity to target DNA, and consequently other characteristics of the systems, such as PAM recognition, cleavage site, and off-target activity. Our results show that it is possible to successfully introduce breaks and gaps in the highly-conserved loop region of crRNAs, and therefore to transform type V-A Cas12a crRNA into a functioning two-component tracrRNA-crRNA-like system analogous to the type II and other type V nucleases (e.g. V-B, V-E). Previous attempt to structurally modify the loop region of CRISPR-Cas12a crRNA in a plasmid-based system resulted in complete termination of gene-cutting efficiency in the presence of AsCas12a nuclease^19^. Their structurally engineered stem duplex was split in the loop region at U (-8), analogous to the structure of our STAR-crRNA modification Split 3. Recently, Shebanova et al. (2022) demonstrated that removal of most of the hairpin scaffold did not significantly affect target DNA cleavage by AsCas12a, FnCas12a, and LbCas12a *in-vitro* and in lysates from human cells. The study showed efficient cleavage caused by AsCas12a charged with high dose of spacer RNA was capable of cleavage of DNA substrates^18^. Contrary, Nguyen et al. (*preprint*) observed limited cleavage efficiency of AsCas12a with truncated crRNA. The study confirmed that most of tested Cas12a orthologs were able to recruit segments of truncated crRNA. Authors failed to verify LbCas12a activity with non-canonical crRNA but observed minor activity with high concentrations of crRNA corresponding to presented here Split 2 and Gap 3. Notably, ErCas12a showed comparable cleaving activity with structures analogous to Split 2 and Gap 3, even at the same concentration^22^. Our findings *in-vitro* and *in-cellulo* demonstrate that Split 2, Split 3 STAR-crRNA, and various other structural modifications to the crRNA loop region have minimal impact on both the DNA cleavage efficiency and on genome editing via HDR in the presence of various Cas12a nucleases. In line with this, we show that the MAD7 nuclease also tolerates the insertion of a 5’ Hairpin structure in addition to the engineered break in the crRNA loop at the position 3, while the addition of a 3’ Hairpin in combination with Split 3 STAR-crRNA reduces the nuclease activity. Furthermore, our findings indicate that the tolerance to such structurally modified crRNAs (STAR and Gap) is both Cas12a nuclease specific as well as dependent upon the location of the disruption within the loop structure and the specific nucleotide at the -10 position. It is important to note that we do not observe any changes in the DNA cleavage site, overhang length, or off-target editing activity of the tested Cas12a nucleases. Finally, these findings give insight into the flexibility of Cas12a nucleases and their tolerance towards crRNA spatial modifications. Together, they advance our understanding of the development of simple design rules for modulating activity and open possibilities for further engineering of CRISPR-Cas12a editing systems. In conclusion, the modularity of STAR-crRNAs offers more flexibility than the wild-type crRNAs, consequently providing a simple engineering approach to dial-up or dial-down the activity. While current autologous cell therapy approaches require high editing efficiencies, reduced on-target activity with eliminated off-target activity would be beneficial for cell line manufacturing, e.g. induced pluripotent stem cell engineering. In addition, STAR-crRNAs may be advantageous in diagnostic tests development, e.g. DETECTR-based diagnostics, and multiplex editing studies for simultaneous targeting of multiple genome loci. Finally, STAR-crRNAs allow for additional modulation level of editing, as well as reduced cost of crRNA synthesis.

While Split 1 STAR-crRNA leads to almost complete termination of MAD7 activity, our findings indicate that nearly entire loop can be removed, except for the ribonucleotide at the position -10, without affecting the nuclease activity. In addition, our data show that all other alterations to the nucleotides in the loop region enable efficient DNA cleavage activity in the presence of Cas12a nucleases and promote efficient gene editing at the immunologically relevant loci in human cells. Crystal structures of Cas12a-crRNA-DNA complexes provide a rationale for the observed activities of split crRNAs used in our study; while Cas12a makes extensive contacts to the crRNA hairpin and DNA complementary sequence, the tetraloop is reported to be solvent-exposed and free of interactions with amino acid residues^31^. Interestingly, the reduced activity of Split 1 may be explained by the reverse Hoogsteen base pairing between U (-10) and A (-18)^31,32^. Evidently, Split 1 STAR-crRNA disrupts the RNA backbone between U (-10) and C (-11), while Split 2 disrupts the backbone between U (-10) and C (-9) and exhibits no loss of activity. This suggests that the positioning of U (-10) adjacent to C (-11) is important for maintaining the reverse Hoogsteen base pair and that this interaction is important for nuclease activity. In contrast, Gao’s team (2016) reported that Cas12a K752 contacts the RNA backbone between G (-6) and U (-7)^31^, at the position of the disruption in Split 5, yet, Split 5 STAR-crRNA exhibits no loss of activity.

Although the classification of CRISPR effector proteins remains unclear^33,34^, and assigning newly discovered nucleases in type V-A may be disputable, all Cas nucleases used in this study are classified as class 2, type V, subtype V-A effectors based on the current classification criteria—single effector proteins guided by a single crRNA while lacking defined tracrRNA in the CRISPR array^25,26^. We show that the SynThetic trAcrRNAs are tolerated by four of the five enzymes tested in this study, while MAD7 and AsCas12a-Ultra (IDT) show comparable activity with the unaltered crRNAs and STAR-crRNAs. In conclusion, our data demonstrate that some of the Cas12a nucleases can utilize split constructs, and as such act analogously to either type II or other type V effectors (e.g. V–B, V–E). Consequently, we observed nuclease-specific differences in the crRNA tolerance, which may inform improved classification criteria and engineering strategies going forward.

## Methods

### Cell culture

Jurkat cells (Leibniz Institute DSMZ-German Collection of Microorganisms and Cell Cultures GmbH (ACC 282)) were grown in RPMI 1640 medium (ThermoFischer Scientific) with 10% heat-inactivated fetal bovine serum (FBS) (ThermoFischer Scientific) supplemented with 1% penicillin–streptomycin antibiotic mix (ThermoFischer Scientific). Cells were grown at 37 °C in 5% CO_2_ incubators at a density of 0.5–1.5 × 10^6^ cells/mL and passaged at 0.1 × 10^6^ cell/mL 24-h before electroporation. For electroporation, cells were harvested by centrifugation (200 × g, RT, 5 min) and resuspended at 10 × 10^6^ cells/mL (2 × 10^5^ cells/20 μL) in supplemented SF nucleofection buffer (Lonza). Cell culture media supernatant was periodically tested for mycoplasma contamination using the MycoAlert PLUS mycoplasma detection kit (Lonza).

### Nuclease expression and purification

*E. coli* BL21 star (DE3) competent cells (ThermoFisher Scientific) were transformed with an expression vector encoding the nuclease gene. 2 × YT medium supplemented with kanamycin was inoculated with a single colony and incubated overnight at 37 °C. The culture was diluted in 1–2 L 2 × YT medium to OD_600_ = 0.1 and grown at 37 °C to OD_600_ = 0.6. At this point, the culture was placed on ice for 15–20 min. Next, IPTG was added in the final concentration of 0.2 mM, and protein expressed overnight (18–20 h) at 18 °C.

Cells were harvested by centrifugation and resuspended in lysis buffer (20 mM Tris, 500 mM NaCl, and 10 mM imidazole, pH = 8.0) supplemented with cOmplete™, EDTA-free protease inhibitor cocktail (Roche). After resuspension, Benzonase® nuclease (Sigma Aldrich, ≥ 250 units/µL, 10 µL per 40 mL lysate) and lysozyme (1 mg/mL lysate) were added and the cell suspension was placed on ice for 30 min. Cells were disrupted on an Avestin EmulsiFlex C-5 homogenizer (15,000—20,000 psi), and insoluble cell debris removed by centrifugation (15,000 g, 4 °C, 15 min).

All subsequent chromatography steps were carried out at 10 °C. The cleared lysate was loaded on a 5-mL HisTrap FF column (GE Healthcare). The resin was washed with 10 column volumes of wash buffer (20 mM Tris, 500 mM NaCl, and 20 mM imidazole, pH = 8.0) and the protein eluted with 10 column volumes of elution buffer (20 mM Tris, 500 mM NaCl, and 250 mM imidazole, pH = 8.0). Fractions containing the protein (typically 13.5 mL) were pooled and diluted to 25 mL in dialysis buffer (250 mM KCl, 20 mM HEPES, and 1 mM DTT, and 1 mM EDTA, pH = 8.0). The sample was dialyzed against 1 L of dialysis buffer at 10 °C using a dialysis membrane tubing with a molecular-weight cut-off of 6–8 kDa (Spectra/Por® standard grade regenerated cellulose, 23 mm wide). The dialysis buffer was replaced after 1–2 h and dialysis continued overnight.

The next day, the dialyzed sample was diluted two-fold in 10 mM HEPES (pH = 8.0) and immediately loaded on a 5-mL HiTrap Heparin HP column (GE Healthcare), pre-equilibrated with buffer A (20 mM Hepes, 150 mM KCl, pH = 8.0). Resin was washed with 2 column volumes of buffer A and the protein eluted using a linear gradient from 0 to 50% of buffer B (20 mM Hepes, 2 M KCl, pH = 8.0) over 12 column volumes. Fractions containing the protein were pooled (typically 10–15 mL) and concentrated to 2 mL using a centrifugal filter unit (Amicon® Ultra-15, 30,000 MWCO; centrifugation at 4 °C). A final chromatography step was performed by injecting the sample on a 120-mL Superdex200 gel filtration column (GE Healthcare) with 50 mM sodium phosphate, 300 mM NaCl, 0.1 mM EDTA, pH = 7.5 as separation buffer. Fractions of interest were pooled and concentrated by centrifugal filtration (Amicon® Ultra-15, 30,000 MWCO; centrifugation at 4 °C) to at least 20 mg/mL (concentration determined by measuring absorbance at 280 nm on a NanoDrop™2000, ThermoFisher) with a percent solution extinction coefficient (Abs 0.1%) of the nuclease). The concentrated protein solution was supplemented with glycerol (20% (v/v) final concentration) and DTT (1 mM final concentration), snap-frozen in liquid nitrogen and stored at -80 °C. Approximately, 20 mg of nuclease was isolated from 1 L of *E. coli* culture.

### STAR crRNA preparation

STAR crRNAs were purchased from IDT, re-suspended in TE buffer (IDTE, IDT) to 100 pmol/µL and prepared by incubating an equimolar mixture of the relevant modulator and targeter RNAs. Sequences are listed in Supplementary Table 1.

### Nuclease search

Following the methodology described in Zetsche et al*.*, 2015, PSI-BLAST program^35^ was used to identify AsCas12a and MAD7 homologs in the NCBI NR database using AsCas12a protein sequence (WP_021736722.1) and MAD7 (WP_055225123.1) as queries with the E-value cut-off of 0.01 with low-complexity filtering and composition-based statistics turned off. The first selection criteria, namely, < 60% sequence similarity to AsCas12a, < 60% sequence similarity to MAD7, and > 80% query coverage, were applied and the results of those searches combined. The dataset was cross-checked to exclude already studied proteins. Multiple sequence’s alignments and pairwise comparisons were constructed using the CLC Main Workbench 7 software (Alignment and Pairwise Comparison with default settings) to exclude proteins of > 90% similarity to already rejected records. The second selection round removed proteins with unknown protein-coding gene or incomplete genomic or chromosomal sequences. Phylogenetic analysis was performed using the Maximum Likelihood Phylogeny (CLC Main Workbench 7.9.1, Neighbor Joining algorithm and Jukes-Cantor Distance measure). DNA sequences coding for selected proteins were collected and analyzed. Genomic data were applied to investigated CRISPR array presence and genomic location of the protein-coding gene using CRISPRCasFinder^36^, CRISPRone^37^, and PILER-CR^38^.

### RNPs formulation

Ribonucleoprotein complexes (RNPs) were generated by incubating relevant crRNAs or STARs with nucleases in molar ratio 3:2 crRNA:nuclease for 10 min at room temperature. For electroporation, the RNP complexes were generated by mixing the specific RNA (150 pmol) and MAD7 (100 pmol), or when indicated, other type V nucleases, in nuclease-free water up to 5 μL. To reduce the complexity and preparation time on the day of the assay^39^, all RNPs were prepared one day before electroporation and stored at 4 °C overnight. Immediately before electroporation, RNPs were incubated for 10 min at room temperature.

### In vitro cleavage assay

Target DNA was amplified from 10 ng wild-type genomic DNA from Jurkat cells using the Phusion High-Fidelity PCR Master Mix with HF Buffer (ThermoFisher Scientific). The PCR products were purified with the Agencourt AMPure XP beads (Ramcon), using the sample to beads ratio of 1:1.8. The DNA was eluted from the beads with nuclease-free water. The RNPs were generated by mixing 1 μL of 12 μM crRNA or STAR with 1 μL of 4 μM nuclease and 10 min incubation at room temperature. The *in vitro* cleavage assay was then performed by adding 200 fmol target DNA in 1 × NEBuffer 2.1 (NEB). The reaction was then incubated for 10 min at 37 °C. The sample was treated with 1 μL Proteinase K (ThermoFisher Scientific) for 10 min at room temperature and the cleavage products analyzed on a 3% agarose gel stained with SYBR safe (ThermoFisher Scientific).

### Electroporation experiments

Lonza 4D Nucleofector with Shuttle unit (V4SC-2960 Nucleocuvette Strips) was used for electroporation, following the manufacturer’s instructions. Jurkat cells were electroporated using the SF Cell Line Nucleofector X Kit (Lonza), CA-137 program, with 2 × 10^5^ cells in 20 µL SF buffer for each nucleofection reaction. The cell suspension was mixed with RNPs, immediately transferred to the nucleocuvette, and subjected to nucleofection in the 96-well Shuttle device. Cells were immediately re-suspended in the cultivation medium and plated on 96-well, flat-bottom, non-cell culture treated plates (Falcon). Cells were harvested 48-h post-transfection for genomic DNA extraction and viability assays. For the **Homology-Directed Repair efficiency assay**, the HDR template, 160 nt long ssDNA (Supplementary Table 2), was collected via pipetting from the HDR plate after the RNPs addition and immediately before the electroporation. The electroporation parameters, cells recovery and proliferation were performed the same way as described above.

### Genomic DNA extraction and PCR amplification

Jurkat cells were harvested by centrifugation (1,000 × g, 10 min) in 96-well, V-bottom plates (Greiner), washed with PBS (Sigma Aldrich) and lysed in 20 μL QuickExctract DNA Extraction Solution (epicentre, Lucigen). DNA was extracted following the manufacturer’s protocol: 15 min at 65 °C, 15 min at 68 °C, 10 min at 95 °C, cooled down and stored at 4 °C. Genomic DNA was diluted 20 × in nuclease-free water before amplicon PCR reactions.

### Targeted amplicon sequencing

Extracted genomic DNA was quantified using the NanoDrop spectrophotometer (ThermoFisher Scientific). Amplicons were constructed in two PCR steps. In the first PCR, regions of interest (150—400 bp) were amplified from 10—30 ng of genomic DNA with primers containing Illumina forward and reverse adapters on both ends (Supplementary Table 3) using Phusion High-Fidelity PCR Master Mix (ThermoFisher Scientific). Amplification products were purified with Agencourt AMPure XP beads (Ramcon), using the sample to beads ratio of 1:1.8. The DNA was eluted from the beads with nuclease-free water and the size of the purified amplicons analyzed on a 2% agarose E-gel using the E-gel electrophoresis system (ThermoFisher Scientific). In the second PCR, unique pairs of Illumina-compatible indexes (Nextera XT Index Kit v2) were added to the amplicons using the KAPA HiFi HotStart Ready Mix (Kapa/Roche). The amplified products were purified with Agencourt AMPure XP beads (Ramcon), using the sample to bead ratio of 1:1.8. The DNA was eluted from the beads with 10 mM Tris–HCl pH = 8.5 + 0.1% Tween20. Sizes of the purified DNA fragments were validated on a 2% agarose gel using the E-gel electrophoresis system (ThermoFisher Scientific), quantified using Qubit dsDNA HS Assay Kit (Thermo Fisher) and then pooled in equimolar concentrations. Quality of the amplicon library was validated using Bioanalyzer, High Sensitivity DNA Kit (Agilent) before sequencing. The final library was sequenced on Illumina MiSeq System using the Miseq Reagent Kit v.2 (300 cycles, 2 × 250 bp, paired-end). De-multiplexed FASTQ files were downloaded from BaseSpace (Illumina).

### NGS data analysis

Initial quality assessment of the obtained reads was performed with FastQC^40^. The sequencing data were aligned and analyzed using CRISPResso2^41^, more specifically CRISPRessoBatch command with the parameters –cleavage_offset 1 -w 10 -wc 1 –expand_ambiguous_alignments. Modification rates from the CRISPResso2 output were analyzed in Excel.

### Equipment and settings

**Gel images** were taken using iBright FL1000 instrument (ThermoFisher Scientific) with following settings: “smart exposure” function was used to set exposure time and avoid overexposure, resolution 1 × 1, optical zoom 1.5, digital zoom 1x, and focus level 385. Images were exported in reverse color. In Fig. 5d, contrast was adjusted for better visibility of the bands. Original images are available in Extended Data Figures.

## Supplementary Information

Supplementary Information 1.

Supplementary Information 2.

Supplementary Information 3.

Supplementary Information 4.

Supplementary Information 5.

Supplementary Information 6.

Supplementary Information 7.

## Data Availability

Next-generation sequencing data have been deposited to the NCBI Sequence Read Archive database under accession PRJNA820998.

## Code availability

No custom code was used in this study that was central to its conclusions.
