# Supplementary material for: CRISPR-Cas12a nucleases function with structurally engineered crRNAs: SynThetic trAcrRNA
Source: Sci Rep. 2022 Jul 16;12:12193. doi: 10.1038/s41598-022-15388-z (PMC9288538; doi:10.1038/s41598-022-15388-z)
Supplement: Supplementary file 2 — Supplementary Information 2. [file 41598_2022_15388_MOESM2_ESM.pdf]

CRISPR-Cas12a nucleases function with structurally engineered crRNAs – SynThetic trAcrRNA

Jedrzejczyk, D. J.[1], Poulsen, L. D.[2], Mohr, M.[1], Damas, N. D.[1], Schoffelen, S.[1], Barghetti, A.[2], Baumgartner, R.[2], Weinert, B. T.[1], Warnecke T.[2*], Gill, R. T.[1,2,**]

[1]Novo Nordisk Foundation Center for Biosustainability, Technical University of Denmark, Kemitorvet 220, 2800 Kongens Lyngby, Denmark

[2]Artisan Bio, 363 Centennial Parkway, Suite 310, Louisville, CO 80027

[*] e-mail: tanya@artisancells.com

[**] e-mail: rtg@artisancells.com, rtg@biosustain.dtu.dk

Supplementary Figure 1

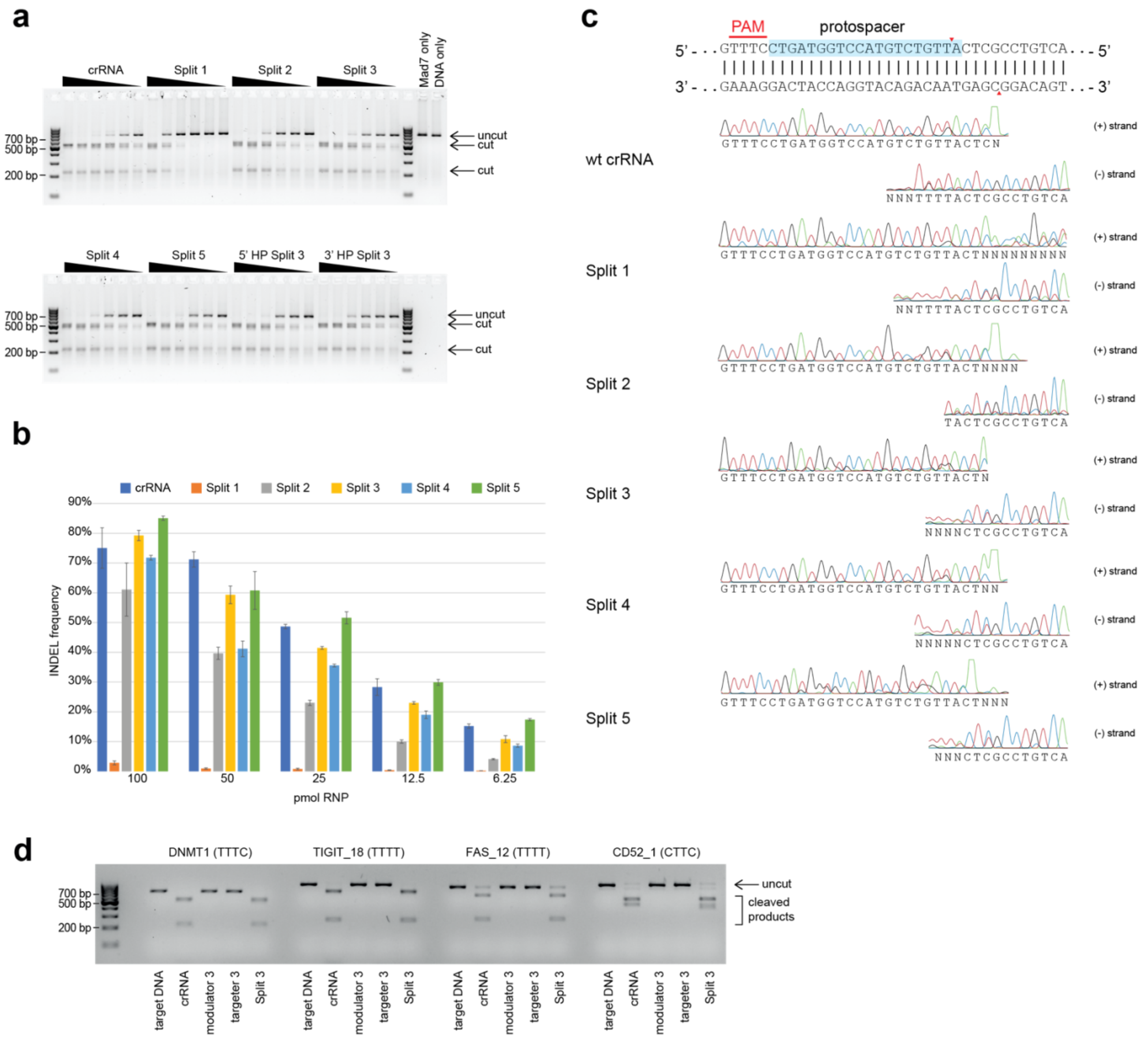

Supplementary Figure 1: **a**, *In-vitro* cleavage assay with dilution series. The amplicon containing the DNMT1 locus target sequence is cleaved with crRNA and STAR-crRNA variants at nuclease : gRNA : target DNA in 20 : 60 : 1, 10 : 30 : 1, 5 :10 : 1, 2.5 : 7.5 : 1, 1.25 : 3.75 : 1, and 0.625 : 1.875 : 1 molar ratio. **b**, Jurkat titration with RNPs assayed by INDEL frequency (%) of MAD7 with wild-type crRNA and Split STAR-crRNAs targeting the DNMT1 locus, measured by amplicon sequencing (error bars: mean ± SD for n = 3). **c**, MAD7 cut site investigation with wild-type crRNA and Split STAR-crRNAs variants, oligonucleotide containing DNMT1 target site was cleaved with RNPs, resulting Sanger sequencing traces are shown. **d**, *in-vitro* cleavage assay on the amplicons containing DNMT1, TIGIT, FAS, and CD52 loci target sites.

Supplementary Figure 2

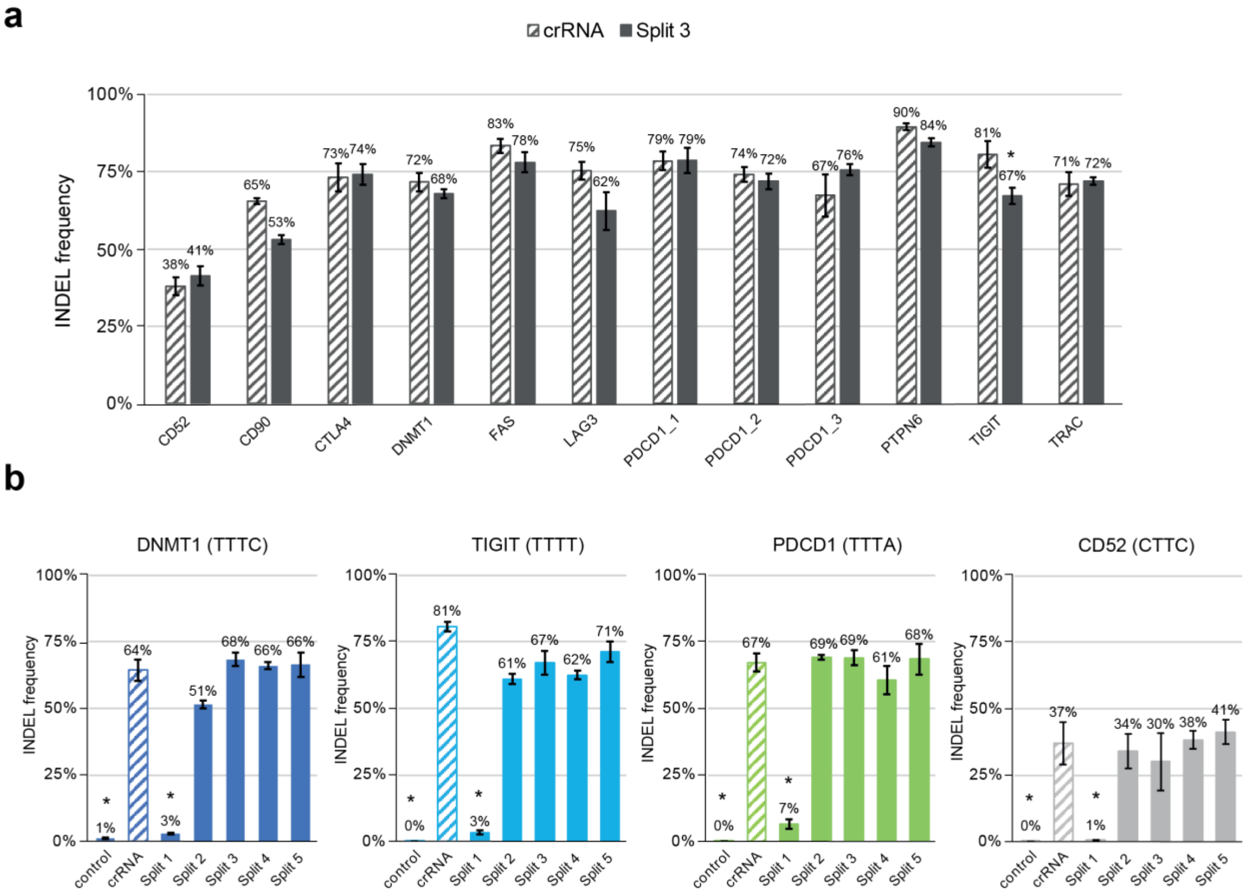

Supplementary Figure 2: **a**, Unnormalized NDEL frequency (%) of MAD7 with wild-type crRNA and Split 3 STAR-crRNA targeting twelve selected loci, measured by amplicon sequencing (error bars: mean ± SEM for n = 6). **b**, Unnormalized INDEL frequency (%) of MAD7 with wild-type crRNA and Split STAR-crRNAs targeting the DNMT1, TIGIT, PDCD1, and CD52 loci with different PAM sequences (TTTC, TTTT, TTTA, and CTTC respectively), measured by amplicon sequencing (error bars: mean ± SEM for n ≥ 3). All samples showing significant changes in INDEL frequencies compared to wild-type crRNAs, $P \leq 0.05$ using two-sided t-test after a global one-way analysis of variance (ANOVA), are indicated with asterisk. The sequences of all crRNAs used are listed in Supplementary Table 1.

Supplementary Figure 3

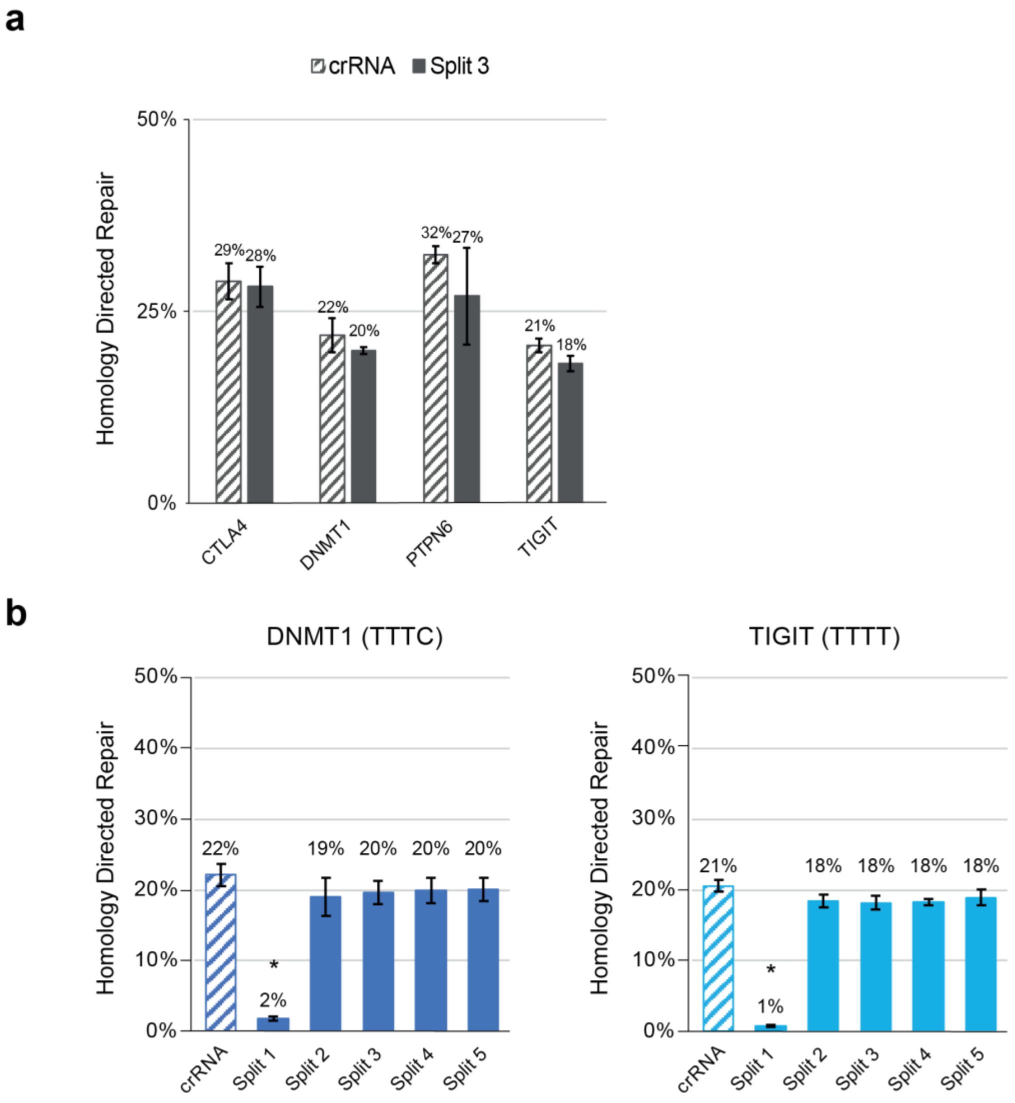

Supplementary Figure 3: **a**, Unnormalized Homology Directed Repair (HDR, %) of MAD7 with wild-type crRNA and Split 3 STAR-crRNA at the CTLA4, DNMT1, PTPN6, and TIGIT loci, measured by amplicon sequencing (error bars: mean ± SEM for n = 6). **b**, Unnormalized Homology Directed Repair (HDR, %) of MAD7 with wild-type crRNA and various Split STAR-crRNA targeting the DNMT1 and TIGIT loci, measured by amplicon sequencing (error bars: mean ± SEM for n = 6). Samples showing significant changes in INDEL frequencies compared to wild-type crRNA, $P \leq 0.05$ using two-sided t-test after a global one-way analysis of variance (ANOVA), are indicated with asterisk. The sequences of all crRNAs used are listed in Supplementary Table 1.

Supplementary Figure 4

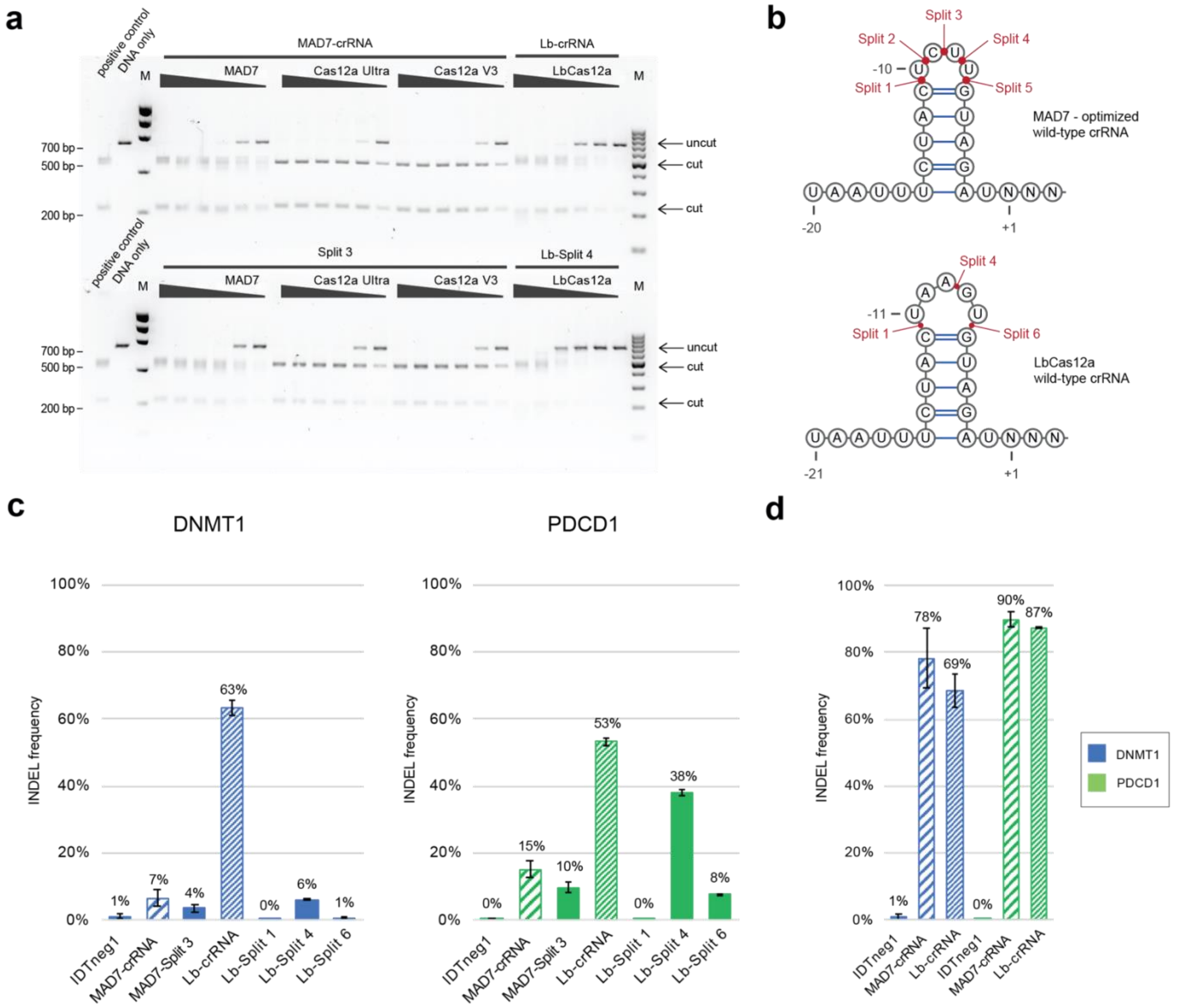

Supplementary Figure 4: **a**, *In-vitro* cleavage assay with dilution series. The amplicon containing the DNMT1 locus target sequence is cleaved with divergent Cas12a nucleases: MAD7, Cas12a Ultra, Cas12a V3 using MAD7 wild-type crRNA and Split 3 STAR-crRNA, and LbCas12a using LbCas12a wild-type crRNA and Lb-Split 4 STAR-crRNA at nuclease : gRNA : target DNA in 20 : 60 : 1, 10 : 30 : 1, 5 :10 : 1, 2.5 : 7.5 : 1, 1.25 : 3.75 : 1, and 0.625 : 1.875 : 1 molar ratio. **b**, Structure of MAD7-optimized crRNA and native LbCas12a crRNA. **c**, INDEL frequency (%) of LbCas12a with MAD7-optimized wild-type crRNA (MAD7-crRNA), MAD7-Split 3 STAR-crRNA, and native Lb-crRNA and Lb-Split 4 STAR-crRNA targeting the DNMT1 and PDCD1 locus, measured by amplicon sequencing (error bars: mean ± SD for n ≥ 3). **d**, INDEL frequency (%) MAD7 with wild-type crRNA (MAD7-crRNA) and native Lb-crRNA targeting the DNMT1 and PDCD1 locus, measured by amplicon sequencing (error bars: mean ± SD for n ≥ 3).
