# Supplementary figures and images for: CRISPR-Cas12a nucleases function with structurally engineered crRNAs: SynThetic trAcrRNA
Source: Sci Rep. 2022 Jul 16;12:12193. doi: 10.1038/s41598-022-15388-z (PMC9288538; doi:10.1038/s41598-022-15388-z)

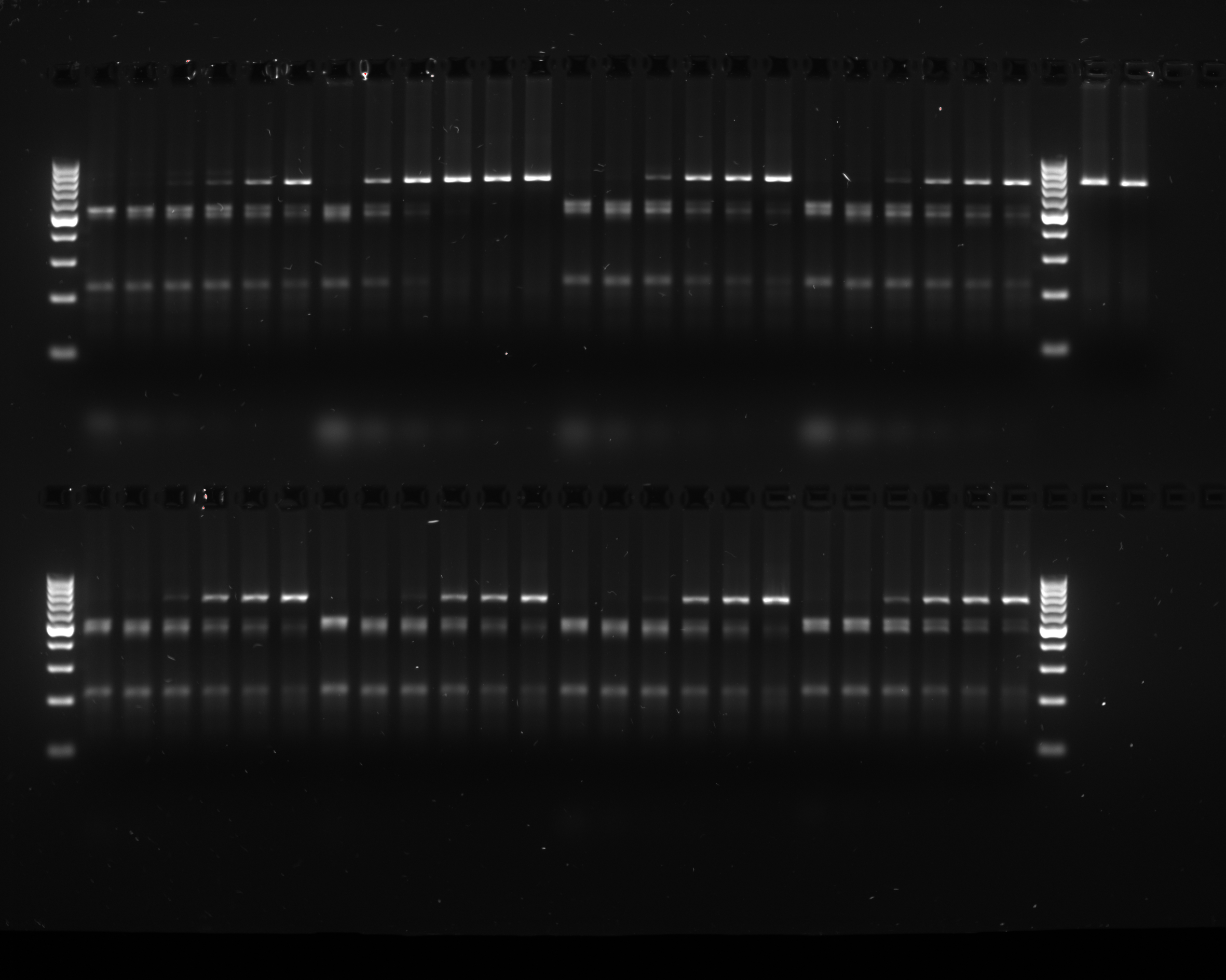

Supplement: Supplementary file 3 — Supplementary Information 3. [file 41598_2022_15388_MOESM3_ESM.tif]

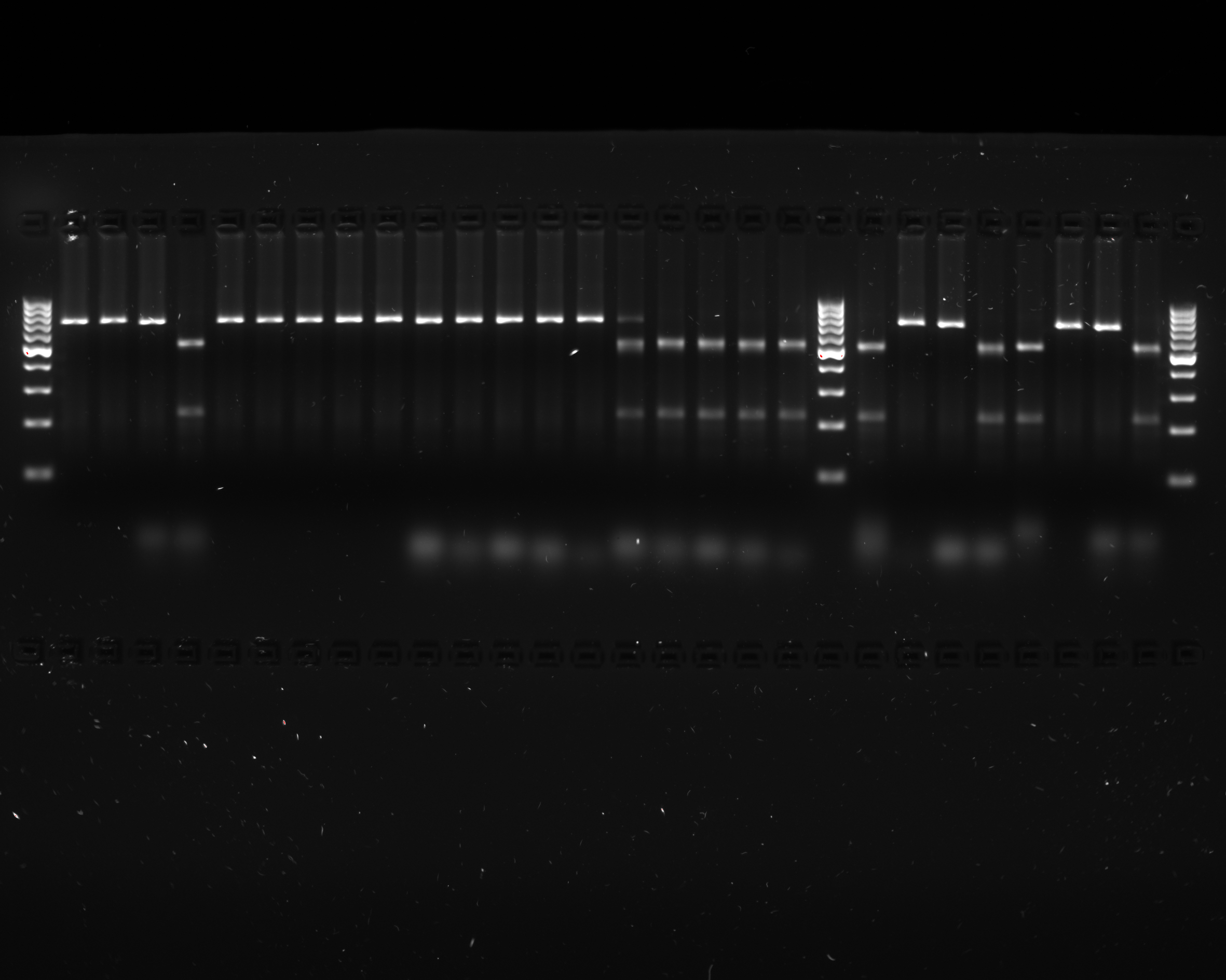

Supplement: Supplementary file 4 — Supplementary Information 4. [file 41598_2022_15388_MOESM4_ESM.tif]

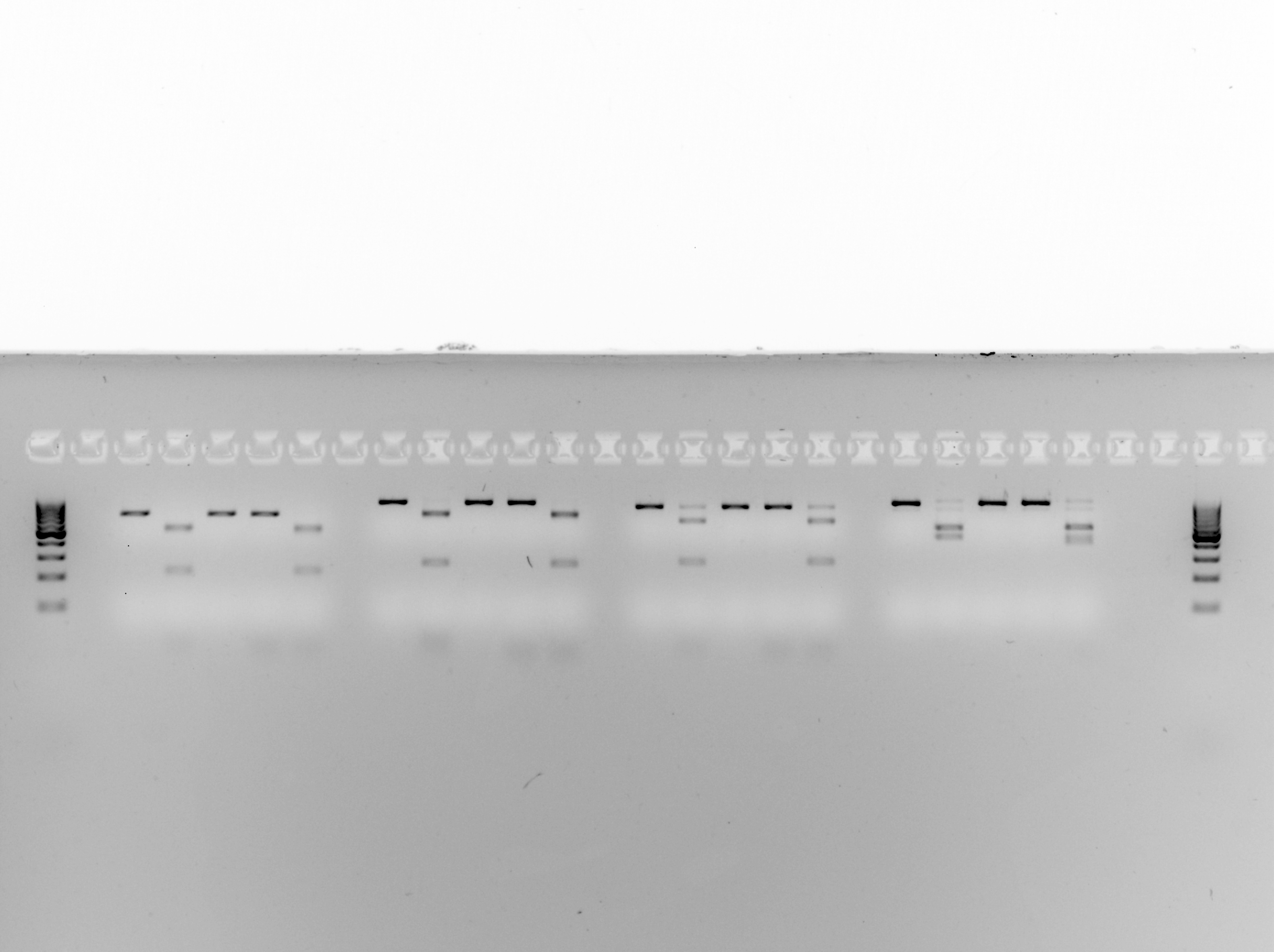

Supplement: Supplementary file 5 — Supplementary Information 5. [file 41598_2022_15388_MOESM5_ESM.png]

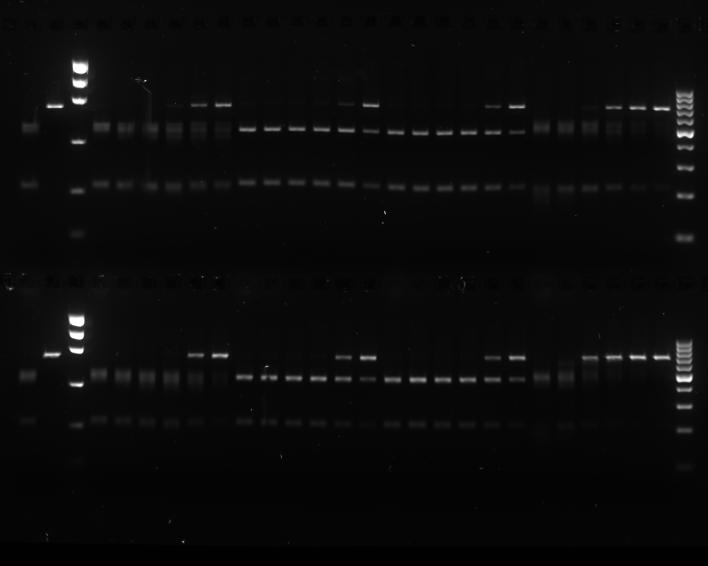

Supplement: Supplementary file 6 — Supplementary Information 6. [file 41598_2022_15388_MOESM6_ESM.tif]

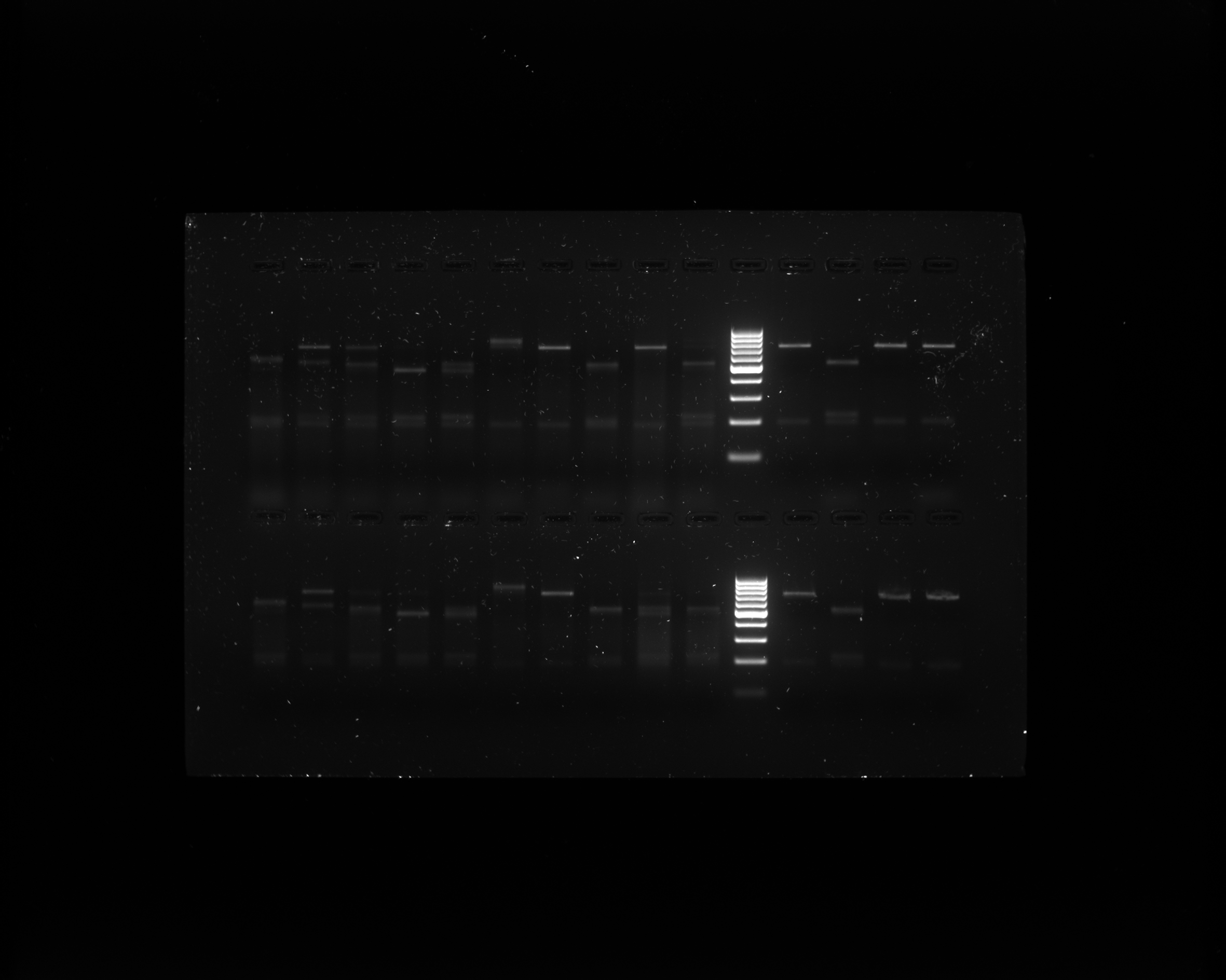

Supplement: Supplementary file 7 — Supplementary Information 7. [file 41598_2022_15388_MOESM7_ESM.tif]
